# Multi-Velocity Encoding Four-Dimensional Flow Magnetic Resonance Imaging in the Assessment of Chronic Aortic Dissection

**DOI:** 10.12945/j.aorta.2017.16.046

**Published:** 2018-09-24

**Authors:** Andrew G. Sherrah, Fraser M. Callaghan, Rajesh Puranik, Richmond W. Jeremy, Paul G. Bannon, Michael P. Vallely, Stuart M. Grieve

**Affiliations:** 1 Sydney Medical School, University of Sydney, Sydney, NSW, Australia; 2 The Baird Institute for Applied Heart and Lung Surgical Research, Sydney, NSW, Australia; 3 Sydney Translational Imaging Laboratory, University of Sydney, Sydney, NSW, Australia; 4 Cardiovascular Magnetic Resonance Sydney, Sydney, NSW, Australia; 5 Institute of Academic Surgery, University of Sydney, Sydney, NSW, Australia; 6 Heart Research Institute, University of Sydney, Sydney, NSW, Australia; 7 Department of Radiology, Royal Prince Alfred Hospital, Sydney, NSW, Australia

**Keywords:** Aorta, Thoracic, Aortic dissection, Magnetic resonance imaging

## Abstract

***Background:*** Chronic descending thoracic aortic dissection (CDTAD) following surgical repair of ascending aortic dissection requires long-term imaging surveillance. We investigated four-dimensional (4D)-flow magnetic resonance imaging (MRI) with a novel multi-velocity encoding (multi-VENC) technique as an emerging clinical method enabling the dynamic quantification of blood volume and velocity throughout the cardiac cycle.

***Methods:*** Patients with CDTAD (n = 10; mean age, 55.1 years; standard deviation (SD) 10.8) and healthy volunteers (n = 9; mean age, 37.1 years; SD 11.4; p < 0.01) underwent 3T MRI, and standard views and 4D-flow data were obtained. Flow measurements were made in selected regions of interest within the ascending and descending thoracic aorta.

***Results:*** The overall flow profile at peak systole was reduced in the false lumen (FL) compared with the true lumen (TL) and normal aortas (p < 0.05 for velocity < 0.4 m/s). Peak systolic flow rate per aortic lumen area (mL/s/cm ^2^ ) was lower in the FL than in the TL (p < 0.05), and both rates were lower than that of control aortas (p < 0.05). Blood flow reversal was higher in the FL than in the TL throughout the descending aorta in CDTAD patients (p < 0.05). A derived pulsatility index was elevated in the TL compared with that in the FL in CDTAD patients. Generated pathline images demonstrated flow patterns in detail, including sites of communication between the true and FL.

***Conclusions:*** 4D-flow MRI revealed FL blood flow and reduced blood flow velocity and flow rate in the TL of CDTAD patients compared with normal aortas of healthy participants. Thus, multi-VENC 4D-flow MRI could serve as an adjunct in the long-term assessment of CDTAD following surgical repair of ascending aortic dissection.

## Introduction

Patients with chronic descending thoracic aortic dissection (CDTAD) following surgical repair of ascending aortic dissection require continued surveillance throughout their lifetime, as some will develop progressive aortic dilatation 1 . The risk of complications can be difficult to predict and appears to be independent of the initial location of dissection (Stanford Type A vs. Type B) or initial medical or surgical therapy 2 . Adverse remodeling of the chronically dissected descending aorta can result in an increased overall diameter > 55 mm 1 , enlargement of the false lumen (FL) 2 , and residual blood flow 3 or partial thrombosis of the FL 4 , which are associated with later complications.

Four dimensional (4D)-flow magnetic resonance imaging (MRI) is an emerging imaging tool that permits accurate quantification of blood flow velocity and volume through the aorta, as well as flow dynamics over time, to be represented as velocity-encoded pathlines 5 6 . The use of a multi-velocity encoding (multi-VENC) approach additionally improves pathline tracking and streamline estimation 7 . Its capability of quantifying bulk flow and measuring flow patterns suggests that 4D-flow MRI may be a useful tool to evaluate CDTAD, particularly for assessment of FL blood flow and intimal flap integrity, providing information beyond current measurements of aortic diameter. Unlike conventional phase-contrast flow MRI, 4D-flow MRI data allow post-acquisition analysis of any region of interest (ROI) in the aorta.

The aim of this study was to assess the potential utility of 4D-flow MRI in measuring true lumen (TL) and FL blood flow in patients with persistent dissection of the descending thoracic aorta following previously surgically repaired ascending aortic dissection. Aortic blood characteristics, including peak velocity, forward flow, reverse flow, and a derived pulsatility index (PI) within the TL and FL, were quantified with 4D-flow MRI and compared with characteristics of healthy control participants without aortic pathology.

## Materials and Methods

### Patients

CDTAD patients were recruited from the Marfan and Aortic Diseases Clinic at Royal Prince Alfred Hospital (RPAH, Sydney, Australia) from January 2014 to June 2015. Patients were included if they were over 18 years of age and had aortic dissection at least 6 months previously. Exclusion criteria were any contraindication to MRI. Healthy control participants were recruited via a flyer advertisement at the hospital and screened by interview for the absence of known aortic or cardiovascular disease.

### MRI Acquisition

Brachial sphygmomanometry was performed immediately following each scan. Data were acquired using a Siemens 3T Skyra MRI (Erlangen, Germany) with electrocardiographic and respiratory gating. All images were analyzed by a radiologist and cardiologist who were accredited in cardiovascular MR. Intravenous contrast was not utilized. All participants underwent a routine cardiac MRI protocol consisting of anatomical and time-resolved (cine) steady-state free precession sequences to confirm the absence of additional cardiac disease or abnormality and to enable placement and acquisition of four-chamber and two-chamber views used in post-processing. Left ventricular ejection fraction (LVEF) was calculated using the Simpson disk summation method. 4D-flow MRI was previously validated against traditional time-resolved phase contrast MRI techniques 8 9 . Scans were obtained using a multi-VENC 4D-flow protocol at three different VENC values of 150, 60, and 20 cm/s covering the entire thoracic aorta 10 . All three scans were isotropic with a spatial resolution of 2.5 mm, and temporal resolution was 16–23 phases per cardiac cycle. Other parameters were a repetition time (TR) of 5.1–5.8 ms and a echo time (TE) of 2.8–3.6 ms. Scan time was approximately 10 min for a VENC of 150 cm/s and 5–6 min for a VENC of 60 and 20 cm/s. The three different VENC datasets were combined on a per-point basis using custom software written in C++, Python, and the VTK Imaging Visualization Toolbox (Kitware Inc., New York). Other acquisition parameters were a flip angle of 8 degrees, acquisition matrix of 144 × 78, and a field of view of 250 × 360 mm. Techniques used for processing the multi-VENC dataset were previously described in detail 7 .

### Data Analysis

Four manually placed transverse planes along the short axis of the thoracic aorta were isolated during post-scan analysis at the levels of the mid ascending aorta (native or prosthetic; Asc _AO_ ) and the midpoints of the proximal, middle, and distal third of the descending thoracic aorta (Prox _AO_ , Mid _AO_ , and Dist _AO_ , respectively). Flow measurements were acquired from manually created intra-luminal ROIs within these planes. Total forward and reverse blood flow volumes through the aortic ROIs were calculated for the entire cardiac cycle (using the R-R interval) as well as peak systolic blood velocity (m/s) and maximal blood flow rate (mL/s). The percentage of flow reversal was determined as reversed volume over total volume. A PI of blood flow was calculated using the following formula 11 : PI = (maximum blood flow (mL/s) – minimum blood flow (mL/s)) / mean blood flow (mL/s).

Images demonstrating blood flow patterns, represented by pathlines (i.e., the path traveled by massless source particles originating from the aortic ROI over a cardiac cycle), were displayed using the Paraview Scientific Visualization Program (Kitware Inc., New York). For CDTAD participants with evident TL and FL fenestrations, additional cross-sectional ROIs were placed perpendicular to these fenestrations for the purpose of flow qualification.

### Statistical Analysis

Statistical analysis was performed using SPSS version 22.0 (IBM, New York). Participant body surface area was calculated as m ^2^ = √ (height (cm) × weight (kg) / 3600) 12 . Normality of continuous data was determined by Shapiro-Wilk tests. Continuous variables are shown as a mean ± standard deviation (SD) when normally distributed or as median and interquartile range (IQR) otherwise. Categorical variables are described as absolute and relative frequencies (percentage). Group differences in baseline data were analyzed using Student’s t-tests, Kruskal-Wallis tests, or Chi-squared tests, as appropriate. Group differences in 4D-flow data (i.e., blood flow velocity, rate, PI, and reversal) were analyzed using Mann-Whitney U tests. The relationship between PI and aortic lumen cross-sectional area was assessed using Spearman’s rank correlation coefficients. A two-tailed p < 0.05 was considered statistically significant.

### Ethics

The study protocol conformed to the ethical guidelines of the 1975 Declaration of Helsinki as reflected by *a priori* approval by the Human Research Ethics Committee of RPAH (Protocol No. X14-0106 and HREC/14/RPAH/129). All participants provided written informed consent.

## Results

### Participant Characteristics

Participants were 10 patients with CDTAD and 9 healthy controls. Demographic characteristics are shown in Table 1 . CDTAD patients were older than control participants (55.1 ± 10.8 years vs. 37.1 ± 11.4 years, p < 0.05). CDTAD patients had experienced dissection of the ascending aorta or aortic arch between 19 months and 16 years prior to study enrollment. All CDTAD patients were on maximal tolerated doses of a beta blocker (n = 3) or combination therapy with an angiotensin II blocker (n = 7), whereas no control participants were on these medications. In CDTAD patients, the underlying aortic pathologies were non-syndromal thoracic aortic aneurysm and dissection (n = 6), hypertension/atherosclerosis (n = 2), Marfan syndrome (n = 1), and iatrogenic aortic dissection secondary to a diagnostic coronary angiogram (n = 1). Both control and CDTAD groups had normal LVEF.

**Table 1.** Characteristics of CDTAD patients and summary of control group.

ID	Age (Years)	Gender	Diagnosis	LVEF (%)	Beta-Blocker Therapy	Ang II Receptor Blocker Therapy	BSA (m ^2^ )	Prior Aortic Surgery	Time to Imaging
**1**	36	Female	MFS	60	Yes	Yes	1.69	AVR and ascending aorta	9 y
**2**	40	Male	ns-TAAD	60	Yes	No	2.05	AVR and ascending aorta	9 y
**3**	52	Male	Iatrogenic dissection	55	Yes	Yes	2.31	Ascending aorta	19 mo
**4**	63	Male	ns-TAAD	55	Yes	Yes	2.06	Ascending aorta	4 y
**5**	57	Male	ns-TAAD	55	Yes	Yes	2.32	AVR and ascending aorta	16 y
**6**	47	Male	ns-TAAD	60	Yes	Yes	2.07	AVR and ascending aorta	3 y
**7**	63	Female	Hypertensive	55	Yes	No	1.99	Ascending aorta and aortic arch	6 y
**8**	69	Male	Hypertensive	60	Yes	Yes	1.90	Ascending aorta	3 y
**9**	56	Male	ns-TAAD	50	Yes	No	2.22	AVR and ascending aorta	14 y
**10**	64	Male	ns-TAAD	55	Yes	Yes	1.91	AVR, ascending aorta and aortic arch	5 y
**CDTAD** **n = 10)**	**55.1** **SD 10.8)**	**2 female,** **8 male**	**-**	**55.0** **55.0–60.0)**	**-**	**-**	**2.1** **SD 0.2)**	**-**	**7.1 y** **SD 4.9)**
**Control (n = 9)**	**37.1** **SD 11.4)** *	**2 female,** **7 male**	**-**	**55.0** **55.0–60.0)**	**None**	**None**	**1.9** **SD 0.2)**	**-**	**-**

* *P* < 0.05 vs. CDTAD. Ang II = angiotensin II; AVR = aortic valve replacement; BSA = body surface area; CDTAD = chronic descending thoracic aortic dissection; MFS = Marfan syndrome; ns-TAAD = non-syndromal thoracic aortic aneurysm and dissection; SD = standard deviation.

The maximum diameter of the descending thoracic aorta was larger in CDTAD patients (39.5, IQR 30.0–43.8 mm) than in control participants (19.0, IQR 17.0–20.0 mm, p < 0.001; Table 2 ). There were no significant differences in intra-luminal area between control participants and the TL of CDTAD patients at any aortic plane. Among CDTAD patients, intra-luminal area was significantly larger in the FL than in the TL.

**Table 2.** Hemodynamic and aortic data for CDTAD patients and summary of control group.

ID	HR (Beats/Min)	SBP (mmHg)	DBP (mmHg)	Maximum Aortic Diameter (mm)	TL Area (cm ^2^ )				FL Area (cm ^2^ )		
					Asc _AO_	Prox _AO_	Mid _AO_	Dist _AO_	Prox _AO_	Mid _AO_	Dist _AO_
**1**	50	108	63	36	5.02	1.48	1.10	1.31	7.53	3.92	3.16
**2**	56	95	51	42	11.67	3.10	1.38	1.38	11.56	10.10	8.55
**3**	78	131	84	26	6.32	5.55	2.46	2.22	-	2.05	1.01
**4**	46	132	76	43	9.34	4.17	2.89	3.17	5.66	5.68	5.23
**5**	73	150	90	46	4.13	3.79	3.34	2.27	5.84	8.14	8.18
**6**	63	129	89	37	10.64	1.58	1.22	1.47	12.11	8.91	7.31
**7**	80	139	79	46	7.72	2.37	1.99	1.54	11.44	9.29	9.82
**8**	41	164	104	28	11.74	5.71	5.14	5.88	-	1.45	-
**9**	60	143	97	23	4.43	7.20	5.67	4.95	-	-	-
**10**	68	130	80	44	7.04	4.90	2.77	2.64	9.54	10.36	9.32
**CDTAD** **n = 10)**	**61.5** **SD 13.4)**	**132.1** **SD 19.7)**	**81.3** **SD 15.6)**	**39.5** **30.0–43.8)**	**7.81** **SD 2.91)**	**3.99** **SD 1.89)**	**2.80** **SD 1.57)**	**2.68** **SD 1.58)**	**9.10** ** (SD 2.76) †**	**6.66** ** (SD 3.47) †**	**6.57** ** (SD 3.15) †**
**Control (n = 9)**	**64.9** **(SD 8.8)**	**118.3** **(SD 9.7)**	**75.6** **(SD 7.3)**	**19.0** **(17.0–20.0)** *	**6.18** **(SD 1.95)**	**3.65** **(SD 0.98)**	**3.32** **(SD 0.81)**	**3.37** **(SD 0.92)**	**-**	**-**	**-**

* *P* < 0.001 vs. CDTAD,

† *P* < 0.05 vs. CDTAD (TL). AscAO = mid ascending aorta; CDTAD = chronic descending thoracic aortic dissection; DBP = diastolic blood pressure; FL = false lumen; HR = heart rate; ProxAO, MidAO, and DistAO = midpoints of the proximal, middle, and distal third of the descending thoracic aorta, respectively; SBP = systolic blood pressure; SD = standard deviation; TL = true lumen.

### Velocity Flow Profile

**Figure 1.**
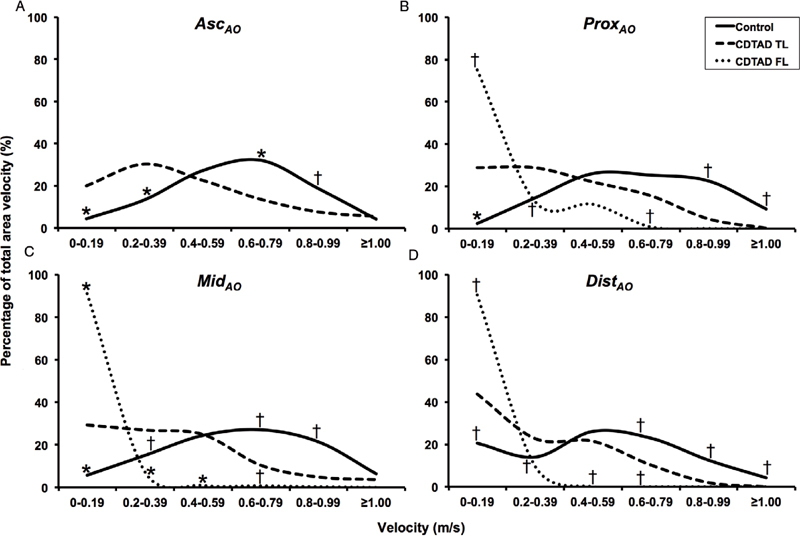
Percentage of total velocity at peak systole stratified by velocity levels and aortic locations. *Panel A* . Asc _AO_ . *Panel B.* Prox _AO_ . *Panel C* . Med _AO_ . *Panel D* . Dist _AO_ . * *p* < 0.001 vs. chronic descending thoracic aortic dissection (CDTAD) true lumen (TL), † *p* < 0.05 vs. CDTAD TL.

In the Asc _AO_ (without aortic dissection), there was a greater proportion by area of low velocity flow (< 0.4 m/s) in the TL of CDTAD patients than in the normal aortas of control patients ( *P* < 0.001; Figure 1A ). The proportions of velocities at mid-range (0.4–0.8 m/s) were similar between groups throughout the descending aorta, whereas the fraction of high velocity flow (> 0.8 m/s) was greater in control participants than in CDTAD patients (p < 0.05 throughout the aorta).

For the Prox _AO_ , Mid _AO_ , and Dist _AO_ in CDTAD patients, the percentage of velocity < 0.4 m/s was significantly higher in the FL than in the TL (all p < 0.05). For both mid-range and high velocities (≥ 0.4 m/s), the relative proportion in the TL was consistently higher than that in the FL, with significant differences at several velocities and aortic locations ( Figure 1B , 1C , and 1D ).

### Proportional Flow and Pulsatility

**Figure 2.**
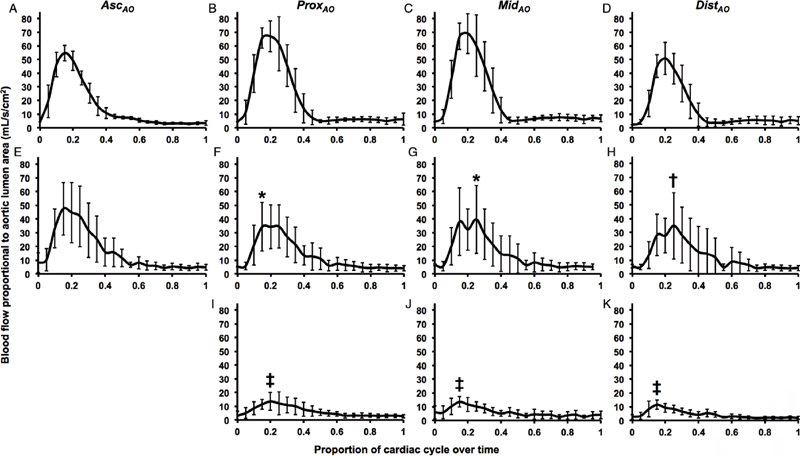
Blood flow rate per aortic lumen area (mL/s/cm ^2^ ) curves standardized by one cardiac cycle. *Panels A-D* . Control participants. *Panels E-H* . TL of CDTAD patients. *Panels I-K* . False lumen (FL) of CDTAD patients. *p < 0.01 vs. control (peak systole), † *p* < 0.05 vs. control (peak systole), ‡ *p* < 0.05 vs. CDTAD TL (peak systole).

Maximal blood flow rate was not significantly different between the normal aortas of control participants and the TL of CDTAD patients at the Asc _AO_ ( *P* = 0.15; Figure 2 ). Maximal flow rate was significantly less in the TL of CDTAD patients ( Figures 2E , 2F , 2G , and 2H ) than in that of control participants ( Figures 2A , 2B , 2C , and 2D ) for all ROIs at the descending thoracic aorta. Within CDTAD patients, proportional maximal blood flow rate in the TL was significantly greater than that in the FL ( Figures 2I , 2J , and 2K ).

**Figure 3.**
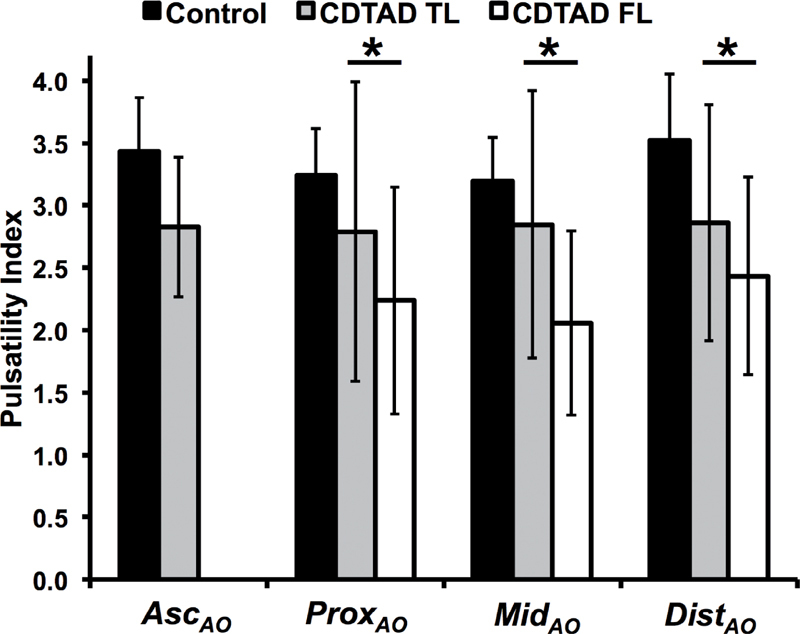
PI of control participants and CDTAD patients. * *P* < 0.05.

A derived PI was compared between groups (Figure 3 ). Between aortic planes, there were no significant differences in PI within groups (all *P* > 0.05). PI was significantly less in the FL than in the TL of CDTAD patients. Across all measured ROIs, PI decreased as aortic lumen area increased, although the correlation was not significant (ρ = -0.4, *P* = 0.7). In only control ROIs, however, this correlation was significant (ρ= -0.36, *P* = 0.03).

### Flow Reversal

At all aortic planes, there were no differences in the percentage of flow reversal between the normal aortas of control participants and the TL of CDTAD patients (Asc _AO_ : 0.9% vs. 2.0%, *P* = 0.3; Prox _AO_ : 1.3% vs. 2.2%, *P* = 0.9; Mid _AO_ : 1.3% vs. 5.7%, *P* = 1.0; and Dist _AO_ : 1.5% vs. 1.6%, *P* = 0.6; respectively). Comparisons between the TL and FL in CDTAD patients revealed significantly lower flow reversal in the TL than in the FL (Prox _AO_ : 2.2% vs. 32.4%, *P* < 0.01; Mid _AO_ : 5.7% vs. 28.6%, *P* < 0.05; and Dist _AO_ : 1.6% vs. 60.0%, *P* < 0.001; respectively).

### Pathlines

**Figure 4.**
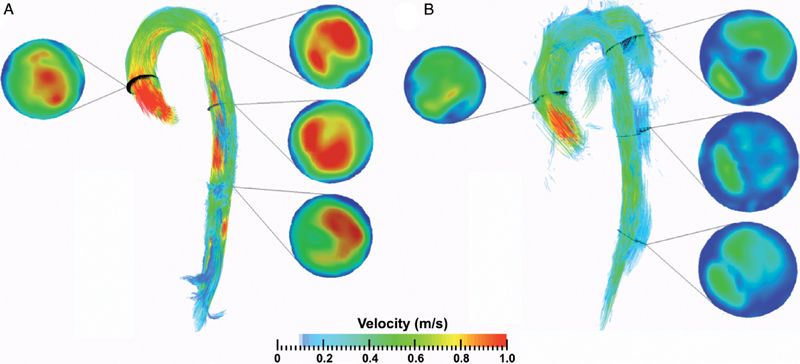
Sagittal pathline views at the isolated thoracic aorta during peak systole within one cardiac cycle. Aortic planes are demonstrated and color-coded by blood flow velocity. *Panel A* . Control participant. *Panel B* . CDTAD patient ID 1 with TL and false lumen FL pathlines isolated (TL sits along the inner curvature of the aortic arch).

**Figure 5.**
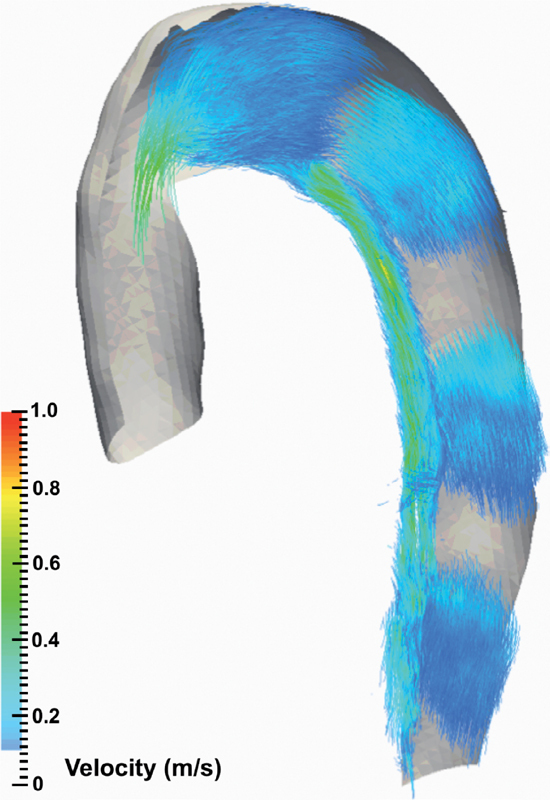
Pathline image at the descending thoracic aorta in CDTAD patient ID 7 demonstrating peak systole within one cardiac cycle. The TL (along the inner curvature of the aortic arch) and FL pathlines are isolated.

**Figure 6.**
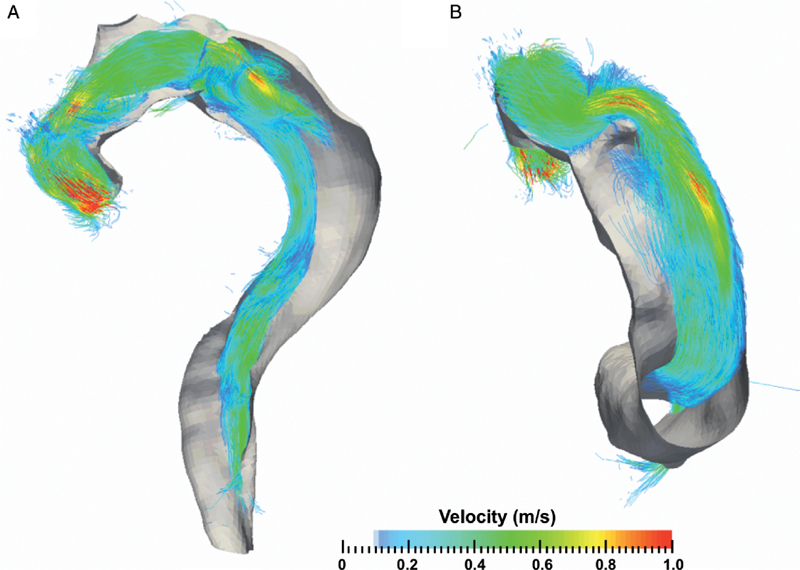
Isolated TL pathline image of the thoracic aorta in CDTAD patient ID 10 at peak systole within one cardiac cycle. The bare volume within the descending aorta represents the extent of the FL. *Panel A* . Sagittal ‘candy cane’ view. *Panel B* . View from caudal aspect along longitudinal plane.

**Figure 7.**
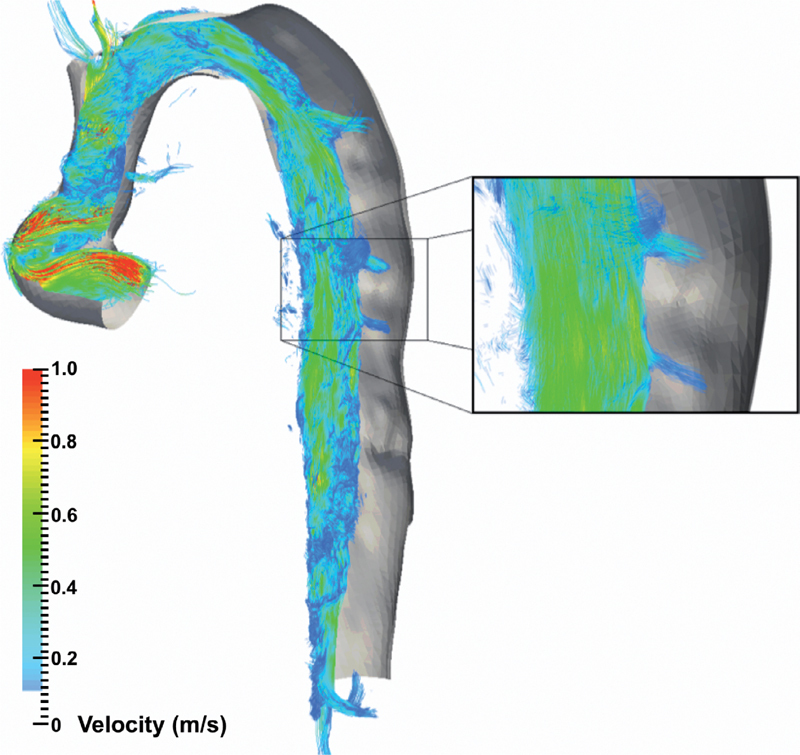
Isolated TL pathline image of the thoracic aorta in CDTAD patient ID 5 at peak systole within one cardiac cycle. The bare volume within the descending aorta represents the extent of the FL. Inset highlights TL and FL communication.

Exemplar illustrations of the use of pathline visualization to better understand abnormal flow dynamics at an individual level are shown in Figures 4 , 5 , 6 , and 7 ). As a normative comparator, the aorta of a control participant is shown in Figure 4A . In Figure 4B , aortic blood flow is shown for CDTAD patient ID 1, who had an entry tear at the proximal descending thoracic aorta into the FL. For CDTAD patient ID 7 ( Figure 5 ), non-laminar blood flow could be visualized at the distal aortic arch at the commencement of a large FL. Within the FL, pathline blood flow did not travel the distance between consecutive aortic planes within one cardiac cycle secondary to low blood velocity. In CDTAD patient ID 10 ( Figure 6 ), pathlines isolated from the ascending aorta and traced into the TL alone similarly demonstrated a relatively large FL. For CDTAD patient ID 5 ( Figure 7 ), at least three distinct communication points were detected and visualized as isolated TL pathline blood flows into a larger FL.

## Discussion

In this study, we demonstrated the potential utility of 4D-flow MRI as a tool in the clinical evaluation of blood flow parameters for patients with CDTAD. 4D-flow MRI was able to quantify differing blood flow characteristics between CDTAD patients and healthy participants. Our unique multi-VENC approach allowed an accurate assessment of FL and TL flow. Current guidelines do not include aortic hemodynamic and flow characteristics as indicators for intervention in CDTAD 1 , as independent of their method of derivation, their relationship with aortic disease progression and physiology remains unclear 13 . Computational fluid dynamic modeling has shown that increased flow and greater wall shear stress are associated with aortic aneurysm expansion in the setting of Type B aortic dissection 14 . Assessment of pulse wave velocity and wall shear stress are among the novel applications of MRI for the measurement of aortic pulsatile flow 15 16 .

### PI as a Predictor of Adverse Events

We used a PI derived from 4D-flow data to characterize flow dynamics within the TL and FL. Although not prognostic for CDTAD, abnormal PI is predictive of aneurysm expansion in porcine models of abdominal aortic aneurysm 17 and in carotid artery aneurysms 11 . PI is inversely proportional to wall shear stress in the vasculature of hypertensive patients 18 , and low wall shear stress is associated with sites of atherogenesis in the aorta as measured by 4D-flow MRI 19 . Elevated PI correlates with increased downstream vascular resistance at other arterial locations, including the pulmonary artery 20 and renal arteries 21 . In CDTAD, greater PI within the FL may be indicative of elevated downstream resistance secondary to thrombosis formation or aortic branch occlusion. We found that PI was reduced throughout the FL, consistent with a chronically dilated lumen with minimal thrombosis and multiple distal exit sites. Among healthy participants, greater PI was associated with reduced aortic lumen area. Although not assessed in our study, the use of after-load reduction medication may also influence the PI. Thus, a derived PI from 4D-flow MRI data may serve as an adjunct to existing predictors of future adverse events in CDTAD.

### Velocity and Flow Profiles in CDTAD

The velocity profile within the FL was markedly dampened compared with that of the TL. Overall blood flow via the FL was significantly less than that via the TL in our CDTAD group, and the transit time of blood via the FL was markedly prolonged with significant blood flow reversal. This was observed despite correction for individual aortic lumen short axis area. These results highlight a particular application of 4D-flow MRI whereby the assessment of aortic blood flow within the TL and FL can be performed separately. This is particularly valuable for the surveillance of CDTAD, in which the distinction between FL thrombosis and slow flow can influence future risk of adverse events 4 .

We found that the percentage of blood flow reversal was significantly higher in the FL than in the TL in CDTAD patients, consistent with previous reports 6 . Additionally, communication between the FL and TL was detected in CDTAD patients. Unlike with conventional MRI-based blood flow assessment, the acquisition of these properties when utilizing 4D-flow is potentially available anywhere within the acquired field of view during post-scan processing.

At the ascending aorta, overall blood velocity was reduced in the control and CDTAD groups despite no significant difference in cross-sectional area. The maximal blood flow rate was not significantly different between groups and likely reflects their normal cardiac function (as measured by LVEF). Patients in the CDTAD group had previously undergone graft replacement of the ascending aorta as well as aortic valve replacement. In our cohort, such prior intervention may have influenced blood velocity but did not appear to influence maximal blood flow rate. Previous investigators of 4D-flow MRI show that wall shear stress and non-laminar blood flow are elevated in this setting following more proximal aortic or valvular surgical intervention 22 .

### Utility of Pathline Analysis

Previous reports using 4D-flow MRI to assess aortic blood flow have included semi-qualitative assessment of blood flow helicity, defined as corkscrew-like movement of encoded pathlines 6 23 . Due to our clinically heterogeneous CDTAD group, we did not formally assess flow helicity. Although helical blood flow is positively correlated with aortic enlargement, its use as a prognostic marker is yet to be confirmed 5 16 . However, our generated pathline images demonstrate additional potential prognosticators, including quantifiable FL blood flow, TL and FL communication, and localized differences in blood flow velocity between normal and chronically dissected aortas. Thus, a multi-VENC approach can allow the differentiation of fast and slow flow domains of the TL and FL.

### Study Limitations

This study has several limitations. CDTAD patients were older than healthy participants, which may have contributed to some of the differences in observed blood flow characteristics. This difference may impact hemodynamics as a result of decreased aortic wall compliance with age. Additionally, our sample sizes were small. Furthermore, CDTAD patients presented with a Stanford Type A aortic dissection, and such patients show a different natural history than patients who initially presented with a Stanford Type B dissection 24 .

## Conclusion

We demonstrate that 4D-flow MRI allows identification of detailed compartmental quantitative blood flow values, including pulsatility, velocity, flow rate, and flow direction, within the TL and FL of CDTAD patients that differ significantly from those of healthy participants. The addition of pathline visualization may allow an improved appreciation of TL and FL hemodynamics, particularly when using a multi-VENC 4D-flow approach. As reliance upon aortic diameter alone as an indicator of intervention is insufficient 25 , 4D-flow MRI could serve as a useful adjunct to the risk stratification of these patients. Longitudinal studies are required to determine the clinical relevance of this imaging modality.
